# Supplementary material for: Neutrophil-to-lymphocyte ratio (NLR), platelet-to-lymphocyte ratio (PLR) are more prominent in retinal artery occlusion (RAO) compared to retinal vein occlusion (RVO)
Source: PLoS One. 2022 Feb 3;17(2):e0263587. doi: 10.1371/journal.pone.0263587 (PMC8812939; doi:10.1371/journal.pone.0263587)
Supplement: Attachment — Submitted filename: Response to Reviewers.docx [file pone.0263587.s002.docx]

RE: PONE-D-21-18118

Title: Neutrophil-to-lymphocyte Ratio (NLR), Platelet-to-lymphocyte Ratio (PLR) are more prominent in retinal artery occlusion (RAO) compared to retinal vein occlusion (RVO).

**Reviewer #1 comments:**

1) RDW is also one of the important inflammation biomarkers. It would be better if authors mention the RDW levels in table 3.

**Response:** We thank the reviewer for the comments. We have added the relevant data about RW and MPV in Table 3, but we didn’t find any significant difference among 3 groups. (page 12, lines 197-200).

2)Did the authors exclude the patients with anemia?

**Response:** We thank the reviewer for the comments. In this study we excluded the patients with any blood disease, including anemia, thrombocytopenia and leukopenia. (pages 6, lines 110).

3) Please add and discuss below references in the discussion part since they are all about your article issue.

1: Kurtul BE, Ozer PA. Neutrophil-to-lymphocyte ratio in ocular diseases: a systematic review. Int J Ophthalmol. 2019 Dec18;12(12):1951-1958. doi:10.18240/ijo.2019.12.18. PMID: 31850181; PMCID: PMC6901879.

2: Kurtul BE, Çakmak Aİ, Elbeyli A, Özarslan Özcan D, Özcan SC, Cankurtaran V.Assessment of platelet-to-lymphocyte ratio in patients with retinal vein occlusion. Ther Adv Ophthalmol. 2020 Nov 18;12:2515841420971949. doi:10.1177/2515841420971949. PMID: 33283155; PMCID: PMC7686591.

3: Elbeyli A, Kurtul BE, Ozcan DO, Ozcan SC, Dogan E. Assessment of Red Cell Distribution Width, Platelet/lymphocyte Ratio,Systemic Immune-inflammation Index, and Neutrophil/lymphocyte Ratio Values in Patients with Central Retinal Artery Occlusion. Ocul Immunol Inflamm. 2021 Sep 15:1-5. doi: 10.1080/09273948.2021.1976219. Epub ahead of print. PMID:34524949.

**Response:** We thank the reviewer for the comments. We have added the reference above in the paper. (references 43,49,50).

4) The study would benefit from minor English editing.

**Response:** We thank the reviewer for the comment. We have done the English editing.

**Reviewer #2 comments:**

Table 1

- Two recent studies, one assessing NLR and PLR role in RVO (Liu et al. Acta Ophthalmol. 2021 Jul 4. doi: 10.1111/aos.14955) and the other investigating CBC measures in RAO (Pinna et al. Acta Ophthalmol. 2021 Sep;99(6):637-643. doi:10.1111/aos.14699) are missing. These should be included and commented.

- The Table should be displayed in a horizontal view. This makes it easier to read.

**Response:** We appreciate the reviewer's comments. Table 1 has been updated to include the two studies. Additionally, we reoriented Table 1 horizontally.

Methods

C-reactive protein and/or erythrocyte sedimentation rate (ESR) are essential to make a diagnosis of arteritic RAO. However, these markers were not analyzed in this paper (line 207). The Authors state that giant cell arteritis was a criterion for exclusion (line 110). How was this exclusion made? If there are no patients with arteritic RAO, all the subjects enrolled in the

study had non-arteritic RAO. If so, this has to be clearly stated throughout the manuscript.

Indeed, if in the RAO group there are patients with both the arteritic and the non-arteritic form, the higher NLR values associated with arteritic RAO may significantly affect the final overall result. This is a critical point to clarify.

**Response:** We thank the reviewer for this great advice. In this study, we have excluded the patients with giant cell arteritis. CRP was tested in only RAO patients. (page 5-6, lines 89-90，109-110).

Discussion

- RAO and RVO are two different vascular retinal disorders with different pathophysiology, risk factors and natural history. What's the rationale for comparing NLR, PLR, and MHR values in these vasculopathies? Please explain.

- line 171. Some recent studies suggest lack of association between NLR and PLR and RVO. Conversely, MPV and, to a lesser extent, RDW may be disease biomarkers in RVO (Pinna et al. Ophthalmic Epidemiol. 2021 Feb;28(1):39-47. doi:10.1080/09286586.2020.1791349). This should be discussed.

**Response:** We thank the reviewer for the comments. Although the pathophysiology of RAO and RVO are not the same, previous studies believed that they were both related to thrombosis and inflammation. Some studies suggest that arterial thrombosis contains more NETs than venous thrombosis. (pages 11, lines 175-184) Although previous studies have shown NLR and/or PLR might be the potential biomarkers in RAO or RVO, we would like to explore if there is some difference between them. We also included the relevant data for RW and MPV in Table 3, but we didn’t find any significant difference among the 3 groups. We speculated that the patients’ race, exclusion criterion and sample size might be responsible for the various results of different studies. (pages 12, lines 197-200).
