# Supplementary material for: Neutrophil-to-lymphocyte ratio (NLR), platelet-to-lymphocyte ratio (PLR) are more prominent in retinal artery occlusion (RAO) compared to retinal vein occlusion (RVO)
Source: PLoS One. 2022 Feb 3;17(2):e0263587. doi: 10.1371/journal.pone.0263587 (PMC8812939; doi:10.1371/journal.pone.0263587)
Supplement: Attachment — Submitted filename: Response to Reviewers.docx [file pone.0263587.s003.docx]

RE: PONE-D-21-18118R1

Title: Neutrophil-to-lymphocyte Ratio (NLR), Platelet-to-lymphocyte Ratio (PLR) are more prominent in retinal artery occlusion (RAO) compared to retinal vein occlusion (RVO).

**Academic Editor comments:**

1) Please incorporate in the Discussion your response to Reviewer #2 comment: "Although the pathophysiology of RAO and RVO are not the same, previous studies believed that they were both related to thrombosis and inflammation. Some studies suggest that arterial thrombosis contains more NETs than venous thrombosis. Although previous studies have shown NLR and/or PLR might be the potential biomarkers in RAO or RVO, we would like to explore if there is some difference between them."

**Response:** We thank the editor for the comments. We have added that in the last paragraph. (page 13, lines 215-218).

2) - Line 125: please change "mean SD" into "mean ± standard deviation (SD) - Line 126: please change "normalcy" into "normality"
- Line 127: please change "the postmortem test" into "post hoc analysis"
- Line 130: please change "to predict" into "predicted"

- Line 152: please move the full stop after (Figure 2)
- Line 200: please change "virous" into "various"
- Line 211: please change "Finally" into "To sum up"
- Line 215: please change "may be a potential biomarker for RAO rather than PLR" into "may be a more reliable biomarker for RAO than PLR"

- Throughout the test: please do not use the short form of auxiliary verbs. Use "we would" instead of "we'd", "did not find" instead of "didn't find", and so on.

**Response:** We thank the editor for the comments. We have done all the revision above.
